# Semi-Kinematic Coupling Design and Analysis for Giant Steerable Science Mirror Prototype of Thirty Meter Telescope

**DOI:** 10.3390/s24113628

**Published:** 2024-06-04

**Authors:** Hongchao Zhao, Wenduo Chen, Qichang An, Peng Guo, Fei Yang

**Affiliations:** 1 School of Advanced Manufacturing, Shenzhen Campus of Sun Yat-sen University, No. 66, Gongchang Road, Guangming District, Shenzhen 518107, China; 2 School of Materials, Shenzhen Campus of Sun Yat-sen University, No. 66, Gongchang Road, Guangming District, Shenzhen 518107, China; 3 Changchun Institute of Optics, Fine Mechanics and Physics, Chinese Academy of Sciences, Changchun 130033, China; anjj@163.com (Q.A.); guopenghust@hotmail.com (P.G.); yangflying@163.com (F.Y.)

**Keywords:** Thirty Meter Telescope, semi-kinematic coupling, repeatability

## Abstract

The Giant Steerable Science Mirror prototype is being developed to assess the tertiary mirror system of the Thirty Meter Telescope. In this study, a new semi-kinematic coupling design is proposed for the prototype based on three pairs of V-grooves and canoe-like components to allow for high repeatability accuracy under heavy loads. A mathematical model was constructed to estimate the repeatability accuracy using the corresponding measurement results and machining errors. The proposed design was verified by an experiment, and the results were consistent with the mathematical model. Furthermore, the results indicate that the repeatability of the semi-kinematic coupling is sufficient for the requirement.

## 1. Introduction

The Thirty Meter Telescope (TMT) is a typical Ritchey–Chrétien optical system, in which all active instruments work on Nasmyth platforms, and is designed to transmit light from 310 to 28,000 nm. However, to relay the optical beams from the secondary mirror to the active instruments of the TMT, the Giant Steerable Science Mirror (GSSM) has been proposed [1,2,3]. This is significantly different from other large telescopes, such as GMT, LAMOST [4], and E-ELT [5]. Thus, frequent maintenance of the tertiary mirror cell assembly (M3CA) is required, owing to unforeseen developments. However, a kinematic coupling could effectively reduce the maintenance cycle of the M3CA. Due to the fact that the tertiary mirror is a flat oval mirror with a major diameter of 4 m, the out-of-plane error needs to be strictly controlled, as it has a significant influence on the telescope [3].

The TMT group has proposed the GSSM prototype (GSSMP) to assess the tertiary mirror system (M3S). This paper presents the design of a semi-kinematic coupling between the M3S and GSSMP. Kinematic couplings are exact constraint design couplings because six known contact points are used to locate one component relative to another [6]. The contact region of kinematic couplings is small, which results in a huge local stress. Therefore, kinematic couplings are frequently applied in low-load working conditions [7,8,9,10,11]. In the M3S, the coupling bears approximately 4.2 tons of loads without inducing a bending moment to the M3CA. Thus, exact constraint design principles or the careful application of elastic averaging should be applied to ensure that components are assembled without causing undue stresses. Reducing deformation and friction on the contact interfaces is the key to achieving a high level of repeatability. However, under the current load, a kinematic coupling would generate contact stress of 1400 MPa, which would cause significant deformation, thus degrading the repeatability of the instruments [12,13].

## 2. Semi-Kinematic Coupling Design

**Figure 1:**
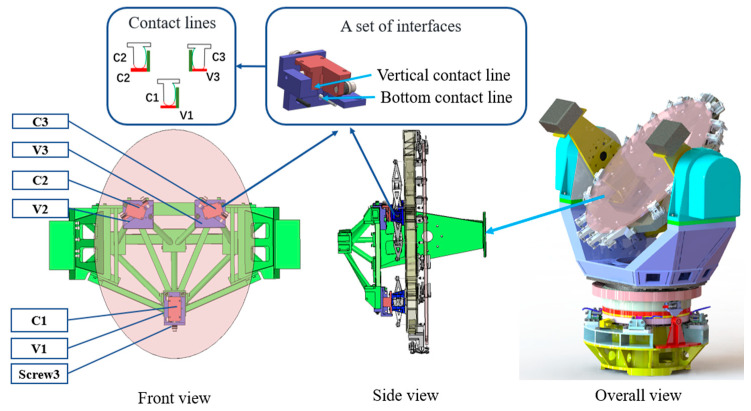
Three-dimensional model of the semi-kinematic coupling.

A classical kinematic coupling mates a triangular configuration of three hemispheres on one assembly to three V-grooves on another assembly, thus enabling essentially the exact constraint of the six degrees of freedom by contacting each other at six small regions. Based on this principle, this study presents a semi-kinematic coupling to solve the conflicts of heavy load and high repeatability accuracy in the M3S. It consists of two sets of components: three V-groove components and three canoe components. The specific configuration and function can be found in Figure 1.

### 2.1. The Coupling Design

**Figure 2:**
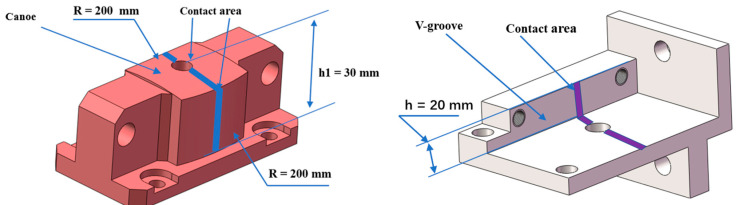
Details of the canoe and V-groove.

Both the bottom and side surface of the canoe are designed to be cylindrical rather than spherical to increase the contact area and reduce the local contact stress, as shown in Figure 2. The contact region is a line, which makes it a semi-kinematic coupling. In the proposed system, the three V-grooves cooperate with the corresponding canoes to identify the position of the M3CA. Furthermore, every pair of canoes and V-grooves can be decoupled using several sets of screws with a spherical gasket. In this design, the radius R of the canoe is 200 mm, and the height h is 20 mm.

### 2.2. Installation Sequence

**Figure 3:**
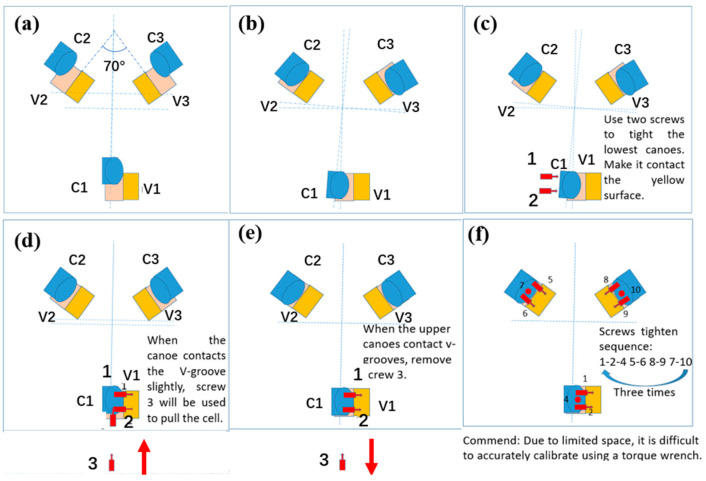
Installation process: (**a**) Components are aligned before installation. (**b**) V2 and C2 as well as V3 and C3 are preliminarily engaged. (**c**) V1 and C1 are engaged slightly by two screws. (**d**) Screw 3 guides the coupling parts into a fine engagement. (**e**) Screw 3 is removed when screws 1 and 2 are tightened. (**f**) Installation is complete.

The installation process is carried out according to the design requirement document. In the GSSMP, when the M3CA prototype is lifted close to the prototype of the M3S positioner assembly, give priority to contact C2 and V2, and then, slowly slide the M3CA prototype until C3 rests on V3. Use screws 1 and 2 to make C1 and V1 contact. Relax the crane, and use screw 3 to guide the M3CA, with a maximum tightening torque of 15 Nm. Then, tighten C1 and V1 using three bolts (5 Nm) before removing screw 3. Fix the remaining two pairs of components with the same sequence. Finally, adjust all screws to 13 Nm ± 1.5 Nm. The detailed installation process can be found in Figure 3. 

### 2.3. Reliability 

Hertz developed a theory for predicting the contact stress between bodies and the term “Hertz contact” to symbolize the high stresses that arise between bodies with in-line contact [14]. An understanding of Hertz contact stresses is thus fundamental to optical designs. Because local Hertz stresses may be high, couplings are often made of hard materials. In practice, the GSSMP mirror cell weighs approximately 92 kg, and 40 Cr is sufficient for the prototype model. 

Preload is the force applied to the coupling to prevent the separation of the contact surfaces. Preload is a critical parameter, which affects repeatability and is proportional to the initial stiffness [15,16]. When the telescope operates in science mode, the load applied on the coupling will vary with the zenith angle. The maximum contact force *f_max_* is approximately 920 N, and the preload *f_pre_* can be calculated as follows:

(1) $$f_{pre}=k_{s}\cdot k_{c}\cdot f_{max}$$

where *k_s_* is the safety factor and *k_c_* is the effective coefficient, which ensures that the interfaces do not separate. Therefore, the maximum preload is 2070 N; however, in actual operation, it is difficult to control the preload accurately. Therefore, a torque wrench is generally used to hold the tightening torque. According to the 50–40–10 rule of the preload screw, the tightening torque must not be less than 13 Nm.

Based on the Hertz contact theory, the maximum contact stress of the kinematic interface would be as follows:

(2) $$\sigma_{\max}=0.418\sqrt{\frac{f_{pre}E}{hR}}=137.8\;\mathrm{MPa}$$

where E = 210 GPa represents the elastic modulus of the material and *f_pre_* is the preload applied onto the contact surface. It is clear that the semi-kinematic design could significantly decrease the stress level. Therefore,

(3) $$\frac{\sigma_{s}}{\sigma_{\max}}\geq 4$$

According to the design requirement document for the tertiary mirror system, the stress in all components that participate in the precision location of the M3 Mirror at limit load shall be maintained below the elastic limit of the component’s material with a factor of safety of 1.2. Obviously, this design can meet this requirement.

## 3. Installation

### 3.1. Component Installation

**Figure 4:**
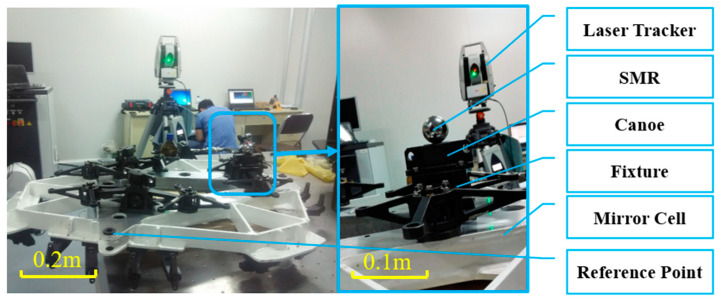
Installation with the laser tracker.

The installation accuracy of the V-grooves and the canoe parts significantly influences the positioning accuracy. A real-time and high-precision detection method can be achieved using laser trackers to control the installation accuracy. To begin, the canoe parts are installed onto the bottom three points of the mirror cell. The positions of the canoe parts are precisely adjusted using the laser tracker and fastened with screws. Then, conical pins are used to secure the canoe parts. When installing the V-grooves, the same process is repeated. After installation, an examination is carried out to verify the installation accuracy, as shown in Figure 4. The measurement results are listed in Table 1.

### 3.2. Installation Process

The coupling is in position when all pairs of contacting surfaces are fully seated, although a slight deviation from the exact center may exist in which the potential energy is minimal. However, uncertainty will be slightly larger in a semi-kinematic coupling than in a kinematic coupling. The factors affecting repeatability accuracy are complex. For example, the profile tolerance of the canoe part should be considered in the semi-kinematic coupling.

It is essential to ensure that the coupling engages. The installation process of the M3CA will involve three translations about [*λ_x_ λ_y_ λ_z_*]′ and three rotations about [*γ_x_ γ_y_ γ_z_*]′. An optimal solution can be found in Section 4.

### 3.3. Error Induced by Friction

Friction is a nonlinear factor that can affect several essential aspects of the design of kinematic couplings. Friction, *f*, may vary in direction and magnitude, which affects the repeatability of the coupling. According to the effect of friction on repeatability accuracy, Hale proposed an estimation method based on the profile and friction coefficient [17,18,19]:

(4) $$\rho \approx \mu {\left(\frac{2}{3R}\right)}^{1/3}{\left(\frac{f_{pre}}{E}\right)}^{2/3}=1.3\;\mu m$$

where *μ* is the friction coefficient. The friction coefficient of the contact surface is 0.38 [20], whereas the actual friction coefficient is 1/2 of the upper limit. Therefore, the repeatability of the positioning error caused by friction is 1.3 μm.

## 4. Simulation

### 4.1. Mathematical Modeling

Exact constraint design can uniquely define the position of a part because the number of contact points equals the degrees of freedom. The semi-kinematic coupling used in this study is based on the same theory as conventional kinematic couplings, making it easier to estimate its performance. The accuracy of the coupling depends on the tolerance and assembly of the components. From the above analysis, the error source comprises the twelve items, as shown in Table 2. 

**Figure 5:**
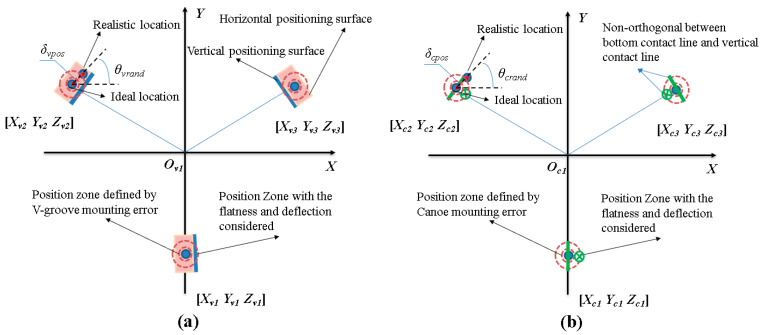
Error sources of the semi-kinematic coupling: (**a**) V-grooves and (**b**) canoes.

As illustrated in Figure 5, the coordinate values of each positioning point for the V-grooves and canoe parts can be determined under random errors based on their relationships and vector geometry mapping. This involves three translations [*λ_x_ λ_y_ λ_z_*] and three rotations [*γ_x_ γ_y_ γ_z_*]. The process of the kinematic interface can be transformed into an optimization problem in mathematics, where the goal is to find an optimal set of translations and rotations. These sets should allow for the x, y, and z positions to cooperate in a manner that minimizes the three-directional error and the L2 norm.

(5) $$\begin{array}{l} \left[\begin{array}{l} X_{vi}\\ Y_{\;vi}\\ Z_{vi} \end{array}\right]=Rz(\beta_{v}RandN()+\varphi_{v}Rand()+w_{vi})\cdot Ry(\frac{\sqrt{2}}{2}\alpha_{v}RandN())\cdot Rx(\frac{\sqrt{2}}{2}\alpha_{v}RandN())\cdot \\ \;\;\;\;\left[\begin{array}{l} X_{vid}+\Delta x_{vi}+\delta_{pos}RandN()\cos(\theta_{rand})+f_{v}RandN()\\ Y_{\;vid}+\Delta y_{vi}+\delta_{pos}RandN()\sin(\theta_{rand})+f_{v}RandN()\\ Z_{vid}+\Delta z_{vi}+f_{v}RandN() \end{array}\right] \end{array}$$

(6) $$\begin{array}{l} \left[\begin{array}{l} X_{ci}\\ Y_{\;\;ci}\\ Z_{ci} \end{array}\right]=Rz(\beta_{c}RandN()+\varphi_{c}Rand()+\gamma_{x}+w_{ci})\cdot Ry(\frac{\sqrt{2}}{2}\alpha_{c}RandN()+\gamma_{y})\cdot Rx(\frac{\sqrt{2}}{2}\alpha_{c}RandN()+\gamma_{z})\cdot \\ \;\;\;\;(\left[\begin{array}{l} X_{cid}+\Delta x_{ci}+\delta_{pos}RandN()\cos(\theta_{rand})+f_{c}RandN()\\ Y_{\;cid}+\Delta y_{ci}+\delta_{pos}RandN()\sin(\theta_{rand})+f_{c}RandN()\\ Z_{cid}+\Delta z_{ci}+f_{s}RandN() \end{array}\right]+\left[\begin{array}{l} \lambda_{x}+\delta_{f}\\ \lambda_{y}+\delta_{f}\\ \lambda_{z}+\delta_{f} \end{array}\right]) \end{array}$$

### 4.2. Repeatability Simulation 

In line with ideal situations, the interface’s solution is unique. However, the outcomes of interface positioning become more complicated when errors are taken into account. As a result, the least squares method is applied to determine the optimal positioning position based on specific errors. The objective function can be expressed as Formula (7).

(7) $$obj={\left(X_{c1}-X_{v1}\right)}^{2}+\sum_{i=2}^{3}{\left[(Y_{ci}-Y_{vi}+{\left(-1\right)}^{i}\cdot \tan(35{}^{\circ})\cdot (X_{ci}-X_{vi}))/\sqrt{1+\tan^{2}(35{}^{\circ})}\right]}^{2}+\sum_{j=1}^{3}{\left(Z_{cj}-Z_{vj}\right)}^{2}$$

**Figure 6:**
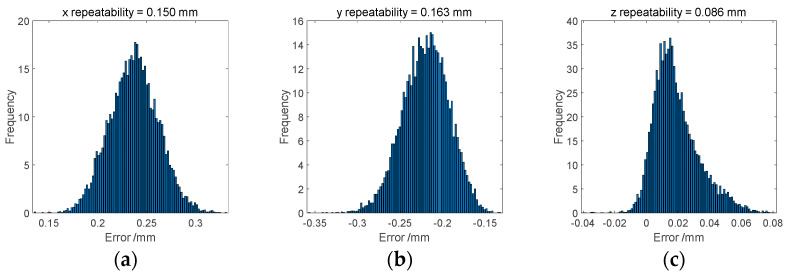
Histogram of semi-kinematic coupling errors: (**a**) *x*-direction, (**b**) *y*-direction, and (**c**) *z*-direction.

Error synthesis simulation is an essential application of the Monte Carlo method. In a typical stochastic simulation, randomness is introduced into simulation models via independent random variables. These random variables are then used as building blocks to simulate more general stochastic systems. In this study, the random variables are listed in Table 2, and the simulation results obtained with the Monte Carlo method in MATLAB R2023a are shown in Figure 6. In them, the repeatability in three directions can be found, which is attributed to machining and assembly errors. The simulation results show that the repeatability values are 0.150 mm, 0.163 mm, and 0.086 mm in the x, y, and z directions, respectively. Obviously, due to the bias error referred to in Table 1, the simulation shows that the error distribution deviates away from the zero point.

### 4.3. Influence on Surface Figure

**Figure 7:**
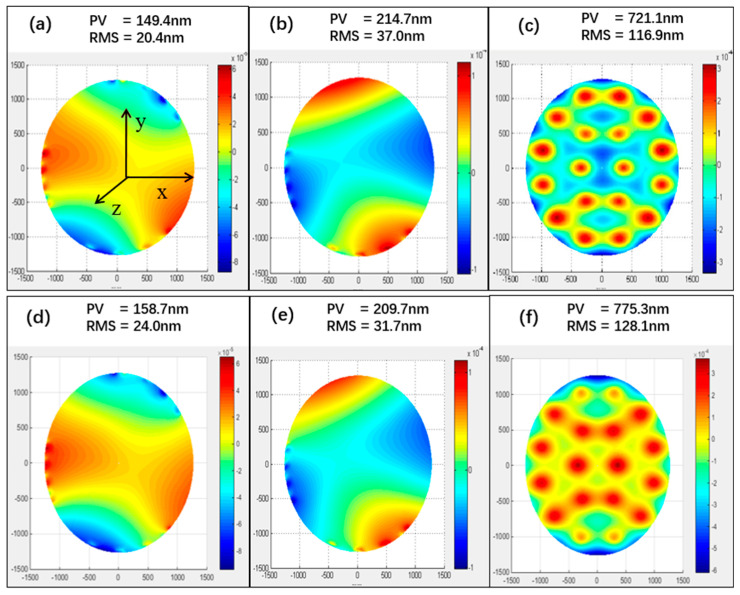
Surface figure: (**a**) Before the installation with gravitational force in the *x*-direction. (**b**) Before the installation with gravitational force in the *y*-direction. (**c**) Before the installation with gravitational force in the *z*-direction. (**d**) After the installation with gravitational force in the *x*-direction. (**e**) After the installation with gravitational force in the *y*-direction. (**f**) After the installation with gravitational force in the *z*-direction.

It is essential to ensure the surface figure of the mirror in the support system design. The tertiary mirror is a solid mirror with uniform thickness. The simplest solution is to maintain the basic principle of the kinematic three-point support system but spread the load supported by each of the three points over a large number of points on the mirror. To effectively improve the system’s stiffness, the rigid center of the coupling should be concentric with the centroid of the payload. Furthermore, the coupling should be designed just under the original three points to prevent the transmission of a bending moment [21,22]. Due to the sizeable radius–thickness ratio, the surface figure is significantly worse when the gravitational force travels in the *z*-direction. Thus, as the contact stiffness is finite, the surface figure will be influenced. A detailed analysis was conducted by FEA method, as shown in Figure 7. In it, the surface figure was slightly degraded in the *x*- and *z*-directions with an improvement in the *y*-direction, and the change was within tolerance.

## 5. Semi-Kinematic Coupling Test

A Leica AT410 laser tracker was used to verify the repeatability of the proposed semi-kinematic coupling. In order to avoid any potential risks, the tertiary mirror was removed from the mirror cell before the experiment was conducted.

### 5.1. Set-Up

**Figure 8:**
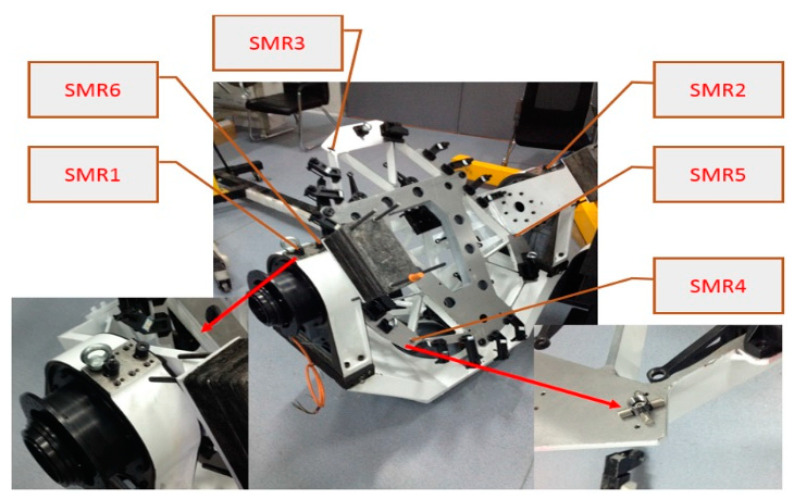
Repeatability test set-up.

Spherically-mounted reflectors (SMRs), also known as corner cubes, are used by laser tracking systems. There are six SMR holders that acted as the measurement points. Holders 1, 2, and 6 monitor oscillation and are mounted on the telescope gimbal. The remaining holders are mounted on the mirror cell and measure the location. The laser tracker is placed such that all SMRs can be detected, as shown in Figure 8.

The measurement process is conducted according to the following procedure. First, the M3CA prototype is installed onto the gimbal, as described in Section 3.1. Then the coordinates of the six points are measured three times. The mirror cell is then removed, and the process is repeated five times.

### 5.2. Measurement

Numerous factors can influence the measurement results; however, three main parts could be identified, namely systematic errors, environmental errors, and other errors. Systematic errors refer to laser tracker errors, including two-axis encoder errors and ranging errors, which increase with the measurement distance. Environmental errors refer to changes in environmental parameters, such as the index of air, temperature, pressure, relative humidity, and vibration. These errors directly change the measurement results. Therefore, improving the measurement accuracy is essential and can be achieved using the following strategy: After each installation, you should wait five minutes before testing to reduce the influence of impacts from the installation. However, the measurement should be performed immediately after five minutes to reduce the air’s effect. During the final data processing, the variation of the tracking frame can be calculated using the fixed three holders. Then, the average locations of the other three holders can be compared to obtain repeatability. 

### 5.3. Results

The average of SMR3, SMR4, and SMR5 may be used to indicate the nominal location coordinates of the mirror cell in accordance with the test configuration. Additionally, the average of SMR1, SMR2, and SMR6 may be used to indicate the location coordinates of the telescope gimble. The amount of movement of the mirror cell with respect to the telescope gimble may thus be written as

(8) $$\Delta Cell_{i}=\left[\begin{array}{l} x_{3i}+x_{4i}+x_{5i}\\ y_{3i}+y_{4i}+y_{5i}\\ z_{3i}+z_{4i}+z_{5i} \end{array}\right]/3-\left[\begin{array}{l} x_{1i}+x_{2i}+x_{6i}\\ y_{1i}+y_{2i}+y_{6i}\\ z_{1i}+z_{2i}+z_{6i} \end{array}\right]/3$$

This experiment is carried out several times. Therefore, the repeatability of the semi-kinematic coupling measurements is given by the following equation:

(9) $$\left[\begin{array}{l} rep_{x}\\ rep_{y}\\ rep_{z} \end{array}\right]=Max{\left(\Delta Cell_{i}\right)}_{i=1\ldots n}-Min{\left(\Delta Cell_{i}\right)}_{i=1\ldots n}=\left[\begin{array}{l} 0.168\\ 0.174\\ 0.064 \end{array}\right]$$

The measurement uncertainty should then be assessed. Measurement data repeatability, which cannot be completely removed but has little impact, is what leads to type A uncertainty. The outcomes are listed below.

(10) $$\left[\begin{array}{l} u_{x1}\\ u_{y1}\\ u_{z1} \end{array}\right]=\frac{Max{\left(\Delta Cell_{i}\right)}_{i=1\ldots n}-Min{\left(\Delta Cell_{i}\right)}_{i=1\ldots n}}{5.3\times \sqrt{5}}=\left[\begin{array}{l} 0.014\\ 0.015\\ 0.005 \end{array}\right]$$

Type B uncertainty caused by the laser tracker can decrease when using a higher precision instrument instead. The initial accuracy of the laser tracker is about 15 μm; however, when the measurement distance is increased by 1 m, the accuracy decreases by 5 μm. Here, the distance is about 2 m. Therefore, the Type B uncertainty can be expressed as follows:

(11) $$U_{2}=\frac{0.015+0.005\times 2}{\sqrt{3}}=0.015$$

Therefore, the combined uncertainty can be obtained by combining Type A and Type B uncertainties.

(12) $$\left[\begin{array}{l} u_{x}\\ u_{y}\\ u_{z} \end{array}\right]=\sqrt{{\left[\begin{array}{l} u_{x1}\\ u_{y1}\\ u_{z1} \end{array}\right]}^{2}+{u_{2}}^{2}}=\left[\begin{array}{l} 0.02\\ 0.02\\ 0.02 \end{array}\right]$$

The results are compared in Table 3. The test results show that the repeatability in the *x*-, *y*-, and *z*-directions is 0.17 mm, 0.17 mm, and 0.06 mm, which is consistent with the mathematical simulation. This indicates that the design is feasible.

## 6. Conclusions

This study proposed a semi-kinematic coupling to solve the high repeatability problem of the interface undergoing heavy loading. With the help of the kinematic positioning principle, the precision positioning function under specific working conditions can be realized by strictly controlling the installation and processing accuracy. Monte Carlo simulation shows that the repeatability of kinematic interfaces in the *x*-, *y*-, and *z*- directions is 0.150 mm, 0.163 mm, and 0.086 mm, respectively. Similarly, test methods were used to test the finished kinematic interfaces, with results showing that the repeatability in the *x*-, *y*-, and *z*- directions reaches 0.17 m, 0.17 mm, and 0.06 mm, respectively, meeting design requirements. The simulation and experiment’s findings are pretty consistent. This proves the viability of the semi-kinematic design.

## Figures and Tables

**Table 1:** Installation errors of the coupling components.

Error	X/mm	Y/mm	Z/mm	W/arcsecond
V1	−0.112	0.182	−0.231	40.2
V2	−0.015	0.084	0.037	32.4
V3	−0.108	0.021	0.127	−30.2
C1	0.064	−0.203	−0.110	15.6
C2	−0.121	−0.088	0.004	36.2
C3	0.124	−0.081	−0.042	−36.8

**Table 2:** Random error sources of semi-kinematic couplings.

	Variable	Errors	Distribution
1	Position error of the interface component *δ*_pos_	0.010 mm	Normal
2	Angle error of the V-grooves *α_v_*	0.002°	Normal
3	Flatness of the V-grooves *f_v_*	0.005 mm	Normal
4	Angle error of the V-grooves induced by installation φv	0.003°	Uniform
5	Angle error of V-grooves induced by the preload *β_v_*	0.006°	Normal
6	Angle error between the two fine surfaces of canoe *α_c_*	0.003°	Normal
7	Profile tolerance of canoe *f_c_*	0.005 mm	Normal
8	Angle error of the Canoe induced by installation *φ_c_*	0.003°	Uniform
9	Angle error of the canoe induced by preload *β_c_*	0.006°	Normal
10	Position error induced by friction *δ_f_*	0.001 mm	Normal
11	Difference between the centers formed by the three canoes and three V-grooves	[*λ_x_ λ_y_ λ_z_*]′	-
12	Angle error between the triangles formed by the tree canoes and the three V-grooves	[*γ_x_ γ_y_ γ_z_*]′	-

**Table 3:** Laser tracker test results.

Repeatability	x	y	z
Requirement	0.200 mm	0.200 mm	0.100 mm
Simulation	0.150 mm	0.163 mm	0.086 mm
Test	0.17 mm ± 0.02 mm	0.17 mm ± 0.02 mm	0.06 mm ± 0.02 mm

## Data Availability

The raw data supporting the conclusions of this article will be made available by the authors on request.
